# Preschool children’s negative affect and social skills in China: The moderating effect of the teacher–child relationship

**DOI:** 10.3389/fpsyg.2022.991039

**Published:** 2022-09-21

**Authors:** Yingjie Wang, Ying Tao, Li Zhu, Yan Li, Dingwen Huang

**Affiliations:** ^1^School of Teacher Education, Huzhou University, Huzhou, China; ^2^Shanghai Early Childhood Education College, Shanghai Normal University, Shanghai, China

**Keywords:** negative affect, social skills, teacher-child relationship, preschool children, China

## Abstract

Negative affect is an important temperament in children, influencing their social skills. However, the evidence for this association in preschool children is limited. Moreover, the underlying mechanisms that explain the relationship between preschool children’s negative affect and social skills remain less understood. Thus, the primary goal of this study was to examine the moderating role of the teacher–child relationship in the associations between negative affect and social skills of Chinese preschoolers. A sample of 198 preschool children (*M*_*age*_ = 58.64 ± 3.83 months, 53% boys), recruited from 13 classes in two public kindergartens in Shanghai, China, participated in this 1-year longitudinal study. The mothers reported children’s negative affect, while the teachers reported children’s social skills and the teacher–child relationship. Results of a series of moderating effect analyses showed that (1) T1 negative affect could significantly negatively predict T2 social skills (cooperation, self-control, and assertion), and (2) the associations between negative affect and social skills were moderated by the teacher–child relationship (including teacher–child closeness and conflict). Specifically, a high level of teacher–child closeness buffered the prediction of negative affect on social skills, while a high level of teacher–child conflict aggravated the said prediction. The findings highlight the importance of the teacher–child relationship in the development of children’s social skills during preschool age when they have a high level of negative affect. This has important implications for the development of interventions to improve teacher–child relationships and children’s social skills.

## Introduction

Social skills refer to a complex set of skills and behaviors that empower an individual to interact appropriately with others and avoid unaccepted responses (Aksoy and Baran, 2010). Preschool children’s social skills can be measured by assessing their cooperation, self-control, and assertion (Gresham et al., 2011; Maleki et al., 2019; Zhu et al., 2022). Cooperation refers to children interacting with others in an organized way, such as helping others, forming friendships, and following social rules. Self-control is defined as children expressing emotions or other social behaviors appropriately in various social situations. Assertion refers to initiating behaviors such as children introducing themselves and seeking information from others.

Preschool children transition from home to kindergarten and social skills are necessary to better adapt to social life, which largely determines their future social adjustment (Zhu Y. T. et al., 2021). A high level of social skills during preschool age can help children internalize social rules, acquire positive social behaviors, and interact with peers effectively (Hosokawa and Katsura, 2017; Hukkelberg et al., 2019). However, deficits in social skills may lead to a series of adaptation problems, such as problem behaviors, loneliness, depression, and even violence (Ziv, 2013; Davis and Qi, 2020; Kamper-DeMarco et al., 2020; García-Fernández et al., 2022). According to the direct linear effects model of children’s development, temperament can directly affect children’s social and emotional development. Some extreme temperaments, such as negative affect and extroversion, can result in poor social adaptation in children (Sanson et al., 2004; Taylor et al., 2014). Thus, it is crucial to explore the influence of the negative affect component of temperament on children’s social skills and determine the key protective and risk factors.

## Negative affect and social skills

Temperament refers to constitutionally based individual differences in reactivity and self-regulation, and it is a core feature that affects personality, emotion, and social behaviors (Rothbart, 2011; Eivers et al., 2012). Among all the aspects of temperament, reactivity refers to the behavioral intensity and frequency of response to stimuli (De Pauw and Mervielde, 2010). It includes two response systems: negative affect and positive affect (Rothbart and Bates, 2006). Negative affect is characterized by irritability, depression, high intensity of negative reactions, and difficulty in appeasement (e.g., anger, fear, frustration, distress owing to one’s limitations, and sadness; Putnam et al., 2008). Children with high levels of negative affect are often accompanied by emotional instability and other adjustment problems, which will directly affect their social interactions, leading to increased behavioral problems, and social maladjustment (Peterson et al., 2018). Negative affect is closely related to children’s poor social adjustment (Taylor et al., 2014; Barone et al., 2019; Acar et al., 2020; Davies et al., 2021). Studies of infants and young children have shown that children with higher levels of anger and frustration in early life tend to exhibit more impulsive and aggressive behaviors and develop poor social relationships and social skills (Jones et al., 2002). Children’s temperament is also closely related to certain sub-social skills. For example, negative affect among infants and toddlers can predict their poor self-regulation (Raikes et al., 2007; Putnam et al., 2008). Qian et al. (2020) investigated two-child families and found that emotionality, activity, reactivity, social inhibition, and concentration among first-born children were significantly correlated with cooperation.

Most of the abovementioned studies have focused on infants and adolescents, and few studies have addressed the relationship between negative affect and social skills in preschool children. Given that preschool years are a critical stage for the development of children’s social interactions and social skills (Kutnick et al., 2007; Maleki et al., 2019), it is meaningful to explore the influence of negative affect on preschool children’s social skills to promote social interactions. In this study, we explored the influence of preschool children’s negative affect on their social skills. Based on the previous studies, we hypothesized that preschool children’s negative affect would be negatively associated with all three indices (cooperation, self-control, and assertion) of their social skills.

## Moderating effect of the teacher–child relationship

Although ample evidence has shown that many children with a high level of negative affect experience a host of social and emotional difficulties, it is also important to stress that not all children with a high level of negative affect will experience problems with social interactions. Several theoretical models have shown that some environmental and individual protective factors can guard against these problems, while certain risk factors can promote them.

According to the developmental systems theory, the development of children’s social skills is influenced by internal (e.g., temperament) and external (e.g., family and school) environmental factors, with children’s internal characteristics dynamically interacting with the external environment for their development (Myers and Pianta, 2008). Since preschool children spend considerable time at daycare, teachers become the primary people at this stage. The attachment theory also stresses that preschool teachers play a crucial role in children’s development during preschool age (Pianta, 1999).

The teacher–child relationship quality refers to the affective nature of children’s interactions with their teachers and can be typically divided into two aspects: teacher–child closeness and teacher–child conflict (Baardstu et al., 2022). Teacher–child closeness is characterized by warm and open communication between teachers and children, which can help children establish a sense of security and belonging, and acts as a protective factor in their development (O’Connor et al., 2011; Verschueren and Koomen, 2012). In contrast, teacher–child conflict is manifested by tense, negative, and hostile communication in the teacher–child relationship, which increases the risk of children’s social maladjustment (Buyse et al., 2011; Coplan et al., 2020). Tensions in the teacher–child relationship also reduce the frequency and quality of teacher–child interactions, thus reducing the opportunities for children to acquire social skills. Some empirical studies have shown that the teacher–child relationship in early childhood years plays an important role in children’s social and emotional competence (Wu et al., 2018; Liu et al., 2020; Paes et al., 2021; Baardstu et al., 2022). For example, Liu et al. (2020) used a cross-lagged analysis and found that toddlers’ teacher–child relationship had a direct effect on their social skills. Baardstu et al. (2022) found that a close teacher–child relationship among 5-year-old children predicted their social competence at 8 years.

There may also be an interaction effect between children’s temperament and the teacher–child relationship on their social skills. That is, the influence of children’s negative affect on their development may be protected by a positive, or intensified by a negative teacher–child relationship. Although few studies have confirmed this conclusion, certain theoretical models or similar studies have suggested that there may be an interaction between the two variables. According to the differential susceptibility model, individuals have different sensitivities to the external environment. Individuals with a susceptible temperament (e.g., negative affect) are more sensitive to both adverse and favorable environments, as compared to their counterparts. Preschool children with a high level of negative affect may perform better in a favorable environment and worse in an adverse environment as compared to typically developing children (Belsky, 1997; Belsky et al., 2007). From this perspective, a conflicted teacher–child relationship is a risk factor that can hinder the development of social skills among children with a high level of negative affect. A close teacher–child relationship may play a moderating role between children’s negative affect and social skills, which can buffer the negative influence of negative affect on children’s social skills. Some empirical studies have revealed similar conclusions. For example, a study of 7-year-old children in Turkey found that highly conflicted student–teacher relationships had a moderating effect on the association between shyness and aggressive behavior. For children with low levels of shyness, the higher level of teacher–child conflict, the more aggressive behaviors they experienced. For children with high levels of shyness, the teacher–child closeness alleviated the maladjustment caused by the shy temperament (Acar et al., 2018). Baardstu et al. (2022) also found that teacher–child closeness had a buffering influence on shy preschool children’s social adjustment difficulties. A study of primary school children found that student–teacher relationships could buffer the negative influence of difficult temperament on their peer interactions (e.g., aggression behaviors and peer victimization; Rudasill et al., 2013). Therefore, it was hypothesized that teacher–child relationships (closeness and conflict) have a moderating effect on the relationship between negative affect and preschool children’s social skills.

## The present study

**FIGURE 1:**
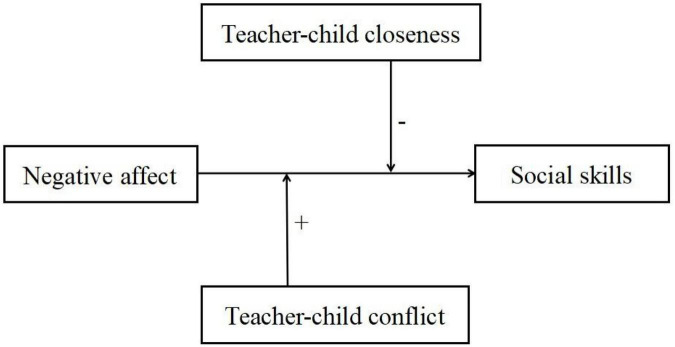
The proposed moderating model.

Based on the relevant theoretical framework and the existing research, it was determined that children’s negative affect and the teacher–child relationship are important factors affecting their social and emotional development, and key interactive effects may exist. However, most prior studies focused on children’s negative affect on their problem behaviors (Crawford et al., 2011; Affrunti and Woodruff-Borden, 2016; Davies et al., 2021), or other aspects of their temperament (e.g., effort control, inhibition) on their social adjustment (Klein et al., 2018; Zhu J. et al., 2021). Few studies have examined the influence of preschool children’s negative affect on their social skills or the underlying mechanisms between them. Against this background, this study aimed to examine the associations between children’s negative affect, social skills, and teacher–child relationships, and the interactions between these variables. Specifically, the following hypotheses and moderating model (Figure 1) were proposed: children’s negative affect will negatively predict their social skills (Hypothesis 1), teacher–child closeness will alleviate the negative prediction of children’s negative affect on their social skills (Hypothesis 2), and teacher–child conflict will intensify the negative prediction of children’s negative affect on their social skills (Hypothesis 3).

## Materials and methods

### Participants

Participants were 198 preschool children recruited from 13 classes in two public kindergartens in Shanghai, China. All children, their mothers, and their teachers took part in this 1-year longitudinal study. The first survey (T1) was conducted in the second semester of the middle class (children aged 4–5 years), and 210 children were recruited. One year later, the second survey (T2) was conducted. Owing to reasons such as school transfers, 12 children could no longer participate in the study. Among the remaining 198 participants, 104 were boys and 94 were girls (T1 mean age = 58.64 ± 3.83 months). Concerning mothers’ education, 13% had a high school diploma, 71% had a bachelor’s degree, and 16% had a master’s degree.

### Measures

#### Negative affect

At T1, the mothers completed the negative affect subscale from the Chinese version of the Child Behavior Questionnaire (Putnam and Rothbart, 2006). The negative affect subscale contains 12 items (e.g., get quite frustrated when prevented from doing something she/he wants to do; α = 0.78). The mothers rated each statement with respect to the extent of their agreement: from 1 (*very appropriate*) to 7 (*very inappropriate*). Higher scores indicate a higher level of children’s negative affect. The negative affect subscale has previously shown satisfactory psychometric properties in the Chinese context (Zhou et al., 2010).

#### Social skills

At T1 and T2, the teachers completed the cooperation, self-control, and assertion subscales from the Chinese version of the Social Skills Rating System-Teacher (Gresham and Elliott, 1990): (1) cooperation (11 items; e.g., participating in games or group activities; α = 0.89), (2) self-control (12 items; e.g., he/she can control his/her emotions when in conflict with others; α = 0.92), and (3) assertion (7 items; e.g., when he/she think the rules are unfair, he/she will challenge the rules appropriately; α = 0.82). The teachers rated each statement from 0 (*never*) to 2 (*often*). Higher scores indicate a higher level of social skills. The overall scale has previously shown satisfactory psychometric properties in the Chinese context (Zhu et al., 2017).

#### Teacher–child relationship

At T2, the teachers completed the teacher–child closeness and teacher–child conflict subscales from the Chinese version of the Student–Teacher Relationship Scale (Pianta and Steinberg, 1992): (1) teacher–child closeness (11 items; e.g., I enjoy an affectionate and warm relationship with the child; α = 0.85) and (2) teacher–child conflict (12 items; e.g., I have a strained relationship with this child; α = 0.87). The teachers rated each statement from 1 (*totally inappropriate*) to 5 (*totally appropriate*). Higher scores indicate a higher level of closeness/conflict. The overall scale has previously demonstrated satisfactory psychometric properties in the Chinese context (Zhang, 2010).

#### Procedure

This study was approved by an appropriate institutional review board. Prior to data collection, approvals from the schools and written informed consent from the parents were obtained. For mothers who agreed to participate, all the questionnaires were distributed to them (*via* their children’s teachers) when they picked up their children from kindergarten. The mothers completed the questionnaires at home and brought them back to the kindergarten the next day. As noted above, data were collected at two-time points: at T1, the mothers reported on children’s negative affect, and the teachers reported on children’s social skills; and at T2, 1 year later, the teachers reported on children’s social skills and the teacher–child relationships. A total of 94% of the original participants remained in the study at T2; thus, the sample attrition rate was considerably low.

### Data analysis

First, correlation and descriptive statistical analyses of the study variables were conducted using Statistical Product and Servcie Solutions (SPSS) 22 (International Business Machine (IBM), Armonk, NY, United States). Second, Hayes’s PROCESS macro (Hayes, 2018; IBM, Armonk, NY, United States) was used to examine the moderating effect of teacher–child relationship in the associations between preschool children’s negative affect and social skills after controlling for children’s gender and social skills at T1. The moderating effect was considered significant when a 95% bias-corrected Confidence intervals (CI) of an interaction term (negative affect × teacher–child relationship) did not contain zero. To further prone the interaction effect, simple slope tests were conducted. The Johnson–Neyman (J–N) technique (Johnson and Fay, 1950) was applied to estimate the region of significance for the simple slope.

## Results

### Preliminary analyses

For all the variables, the rate of missing data ranged from 0.07 to 1.5%. Little’s Missing Completely at Random (MCAR) test showed that the missing data were completely at random [*χ^2^*(73) = 1.12, *p* = 0.23]. The missing data were imputed using the Expectation Maximization (EM) algorithm (Graham, 2009). The descriptive statistics and correlations are shown in Table 1. Children’s gender (0 = boy, 1 = girl) was significantly and positively related to T1 cooperation, T1 self-control, T1 assertion, and T2 self-control, which indicated that girls had a higher level of social skills than boys. T1 cooperation, T1 self-control, and T1 assertion were significantly and positively related to the three aspects of social skills at T2. As such, children’s gender and the three aspects of social skills at T1 were input as control variables in the following moderation analysis. Children’s negative affect was significantly and negatively related to T2 cooperation, T2 self-control, and T2 assertion. Teacher–child closeness was significantly and positively related to the three aspects of social skills at T1 and T2. Teacher–child conflict was significantly and negatively related to T2 social skills.

**TABLE 1:** Correlations among study variables.

	1	2	3	4	5	6	7	8	9	10
1. Gender	–									
2. T1 Negative affect	–0.06	–								
3. T1 Cooperation	0.21**	–0.09	–							
4. T1 Self-control	0.19**	–0.03	0.76***	–						
5. T1 Assertion	0.15*	–0.11	0.90***	0.67***	–					
6. T2 Cooperation	0.10	−0.21**	0.24***	0.15*	0.20**	–				
7. T2 Self-control	0.19**	−0.19**	0.22**	0.25***	0.19**	0.79***	–			
8. T2 Assertion	0.09	−0.19**	0.18**	0.10	0.19**	0.89***	0.78***	–		
9. T2 Teacher-child closeness	0.08	0.02	0.30***	0.21**	0.26***	0.38***	0.29***	0.40***	–	
10. T2 Teacher-child conflict	–0.10	–0.02	0.06	0.08	0.11	−0.24**	−0.31***	−0.23**	0.03	–
*M*	–	3.74	1.46	1.47	1.42	1.59	1.61	1.55	3.90	2.04
*SD*	–	0.78	0.46	0.47	0.48	0.44	0.43	0.48	0.61	1.17

Gender 0 = boy, 1 = girl; *p < 0.05, **p < 0.01, ***p < 0.001.

### Moderating effects of teacher–child relationship

Children’s gender and social skills at T1 were included as control variables, and all the study variables were standardized before they were entered into the moderating models. First, the direct effects of negative affect on the three aspects of social skills (Tables 2–4) were consistent with Hypothesis 1: preschool children’s negative affect significantly negatively predicted cooperation (β = −0.24, *t* = −3.68, *p* < 0.001, *95% CI* [−0.37, −0.11]), self-control (β = −0.2, *t* = −2.94, *p* < 0.01, *95% CI* [−0.33, −0.07]), and assertion (β = −0.22, *t* = −3.29, *p* < 0.001, *95% CI* [−0.35, −0.09]).

**TABLE 2:** Moderating effect of negative affect and teacher-child relationship on cooperation.

Outcome variables	Predictors	β	*SE*	*t*	*95% CI*
T2 Cooperation	Gender	0.02	0.14	0.15	[−0.25, 0.29]
	T1 Cooperation	0.20	0.07	2.87**	[0.06, 0.34]
	T1 Negative affect	–0.24	0.07	−3.68***	[−0.37, −0.11]
	T2 Teacher-child closeness	0.33	0.07	4.62***	[0.19, 0.47]
	T1 Negative affect × T2 Teacher-child closeness	0.18	0.08	2.28*	[0.02, 0.34]
T2 Cooperation	Gender	0.01	0.14	0.01	[−0.28, 0.28]
	T1 Cooperation	0.27	0.07	3.82***	[0.13, 0.40]
	T1 Negative affect	–0.27	0.07	−3.83***	[−0.40, −0.13]
	T2 Teacher-child conflict	–0.25	0.07	−3.41***	[−0.40, −0.11]
	T1 Negative affect × T2 Teacher-child conflict	–0.17	0.08	−2.19*	[−0.33, −0.02]

*p < 0.05, **p < 0.01, ***p < 0.001.

**TABLE 3:** Moderating effect of negative affect and teacher-child relationship on self-control.

Outcome variables	Predictors	β	*SE*	*t*	*95% CI*
T2 Self-control	Gender	0.22	0.14	1.56	[−0.06, 0.49]
	T1 Self-control	0.23	0.07	3.18**	[0.09, 0.38]
	T1 Negative affect	–0.20	0.07	−2.94**	[−0.33, −0.07]
	T2 Teacher-child closeness	0.23	0.07	3.25**	[0.09, 0.37]
	T1 Negative affect × T2 Teacher-child closeness	0.15	0.08	1.86	[−0.01, 0.31]
T2 Self-control	Gender	0.17	0.14	1.26	[−0.10, 0.44]
	T1 Self-control	0.28	0.07	4.02***	[0.14, 0.41]
	T1 Negative affect	–0.22	0.07	−3.28**	[−0.35, −0.09]
	T2 Teacher-child conflict	–0.33	0.07	−4.60***	[−0.47, −0.19]
	T1 Negative affect × T2 Teacher-child conflict	–0.16	0.08	−2.08*	[−0.31, −0.01]

*p < 0.05, **p < 0.01, ***p < 0.001.

**TABLE 4:** Moderating effect of negative affect and teacher-child relationship on the assertion.

Outcome variables	Predictors	β	*SE*	*t*	*95% CI*
T2 assertion	Gender	0.01	0.13	0.06	[−0.26, 0.27]
	T1 assertion	0.17	0.07	2.44*	[0.03, 0.30]
	T1 negative affect	–0.22	0.07	−3.29**	[−0.35, −0.09]
	T2 teacher-child closeness	0.36	0.07	5.07***	[0.22, 0.50]
	T1 negative affect × T2 teacher-child closeness	0.20	0.08	2.49*	[0.04, 0.36]
T2 assertion	Gender	–0.01	0.14	–0.02	[−0.28, 0.28]
	T1 assertion	0.24	0.07	3.39***	[0.10, 0.37]
	T1 negative affect	–0.24	0.07	−3.46**	[−0.38, −0.10]
	T2 teacher-child conflict	–0.26	0.07	−3.53***	[−0.41, −0.12]
	T1 negative affect × T2 teacher-child conflict	–0.18	0.08	−2.31*	[−0.34, −0.03]

*p < 0.05, **p < 0.01, ***p < 0.001.

Second, consistent with Hypotheses 2 and 3, a series of interactions between negative affect and teacher–child relationship (closeness and conflict) predicted children’s social skills (Tables 2–4). In each of the relevant models, after controlling for children’s gender and the three aspects of social skills at T1, we found a significant interaction of negative affect × teacher–child closeness on cooperation (β = 0.18, *t* = 2.28, *p* < 0.05, *95% CI* [0.02, 0.34]) and assertion (β = 0.2, *t* = 2.49, *p* < 0.05, *95% CI* [0.04, 0.36]). We also found a significant interaction of negative affect × teacher–child conflict on cooperation (β = −0.17, *t* = −2.19, *p* < 0.05, *95% CI* [−0.33, −0.02]), self-control (β = −0.16, *t* = −2.08, *p* < 0.05, *95% CI* [−0.31, −0.01]), and assertion (β = −0.18, *t* = −2.31, *p* < 0.05, *95% CI* [−0.34, −0.03]).

**FIGURE 2:**
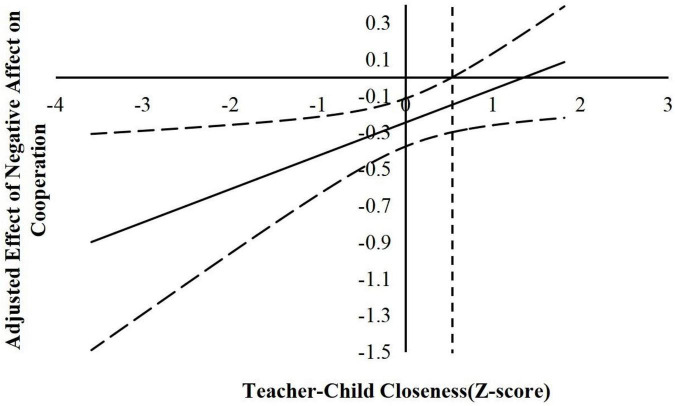
The moderating effect of teacher-child closeness in the prediction of children’s negative affect on cooperation. The dashed vertical line indicates the point along the teacher-child closeness in which the negative affect regression coefficient transitions from significance (left of the dashed vertical line) to non-statistical significance (right of the dashed vertical line). The value of the dashed vertical line was 0.53.

**FIGURE 3:**
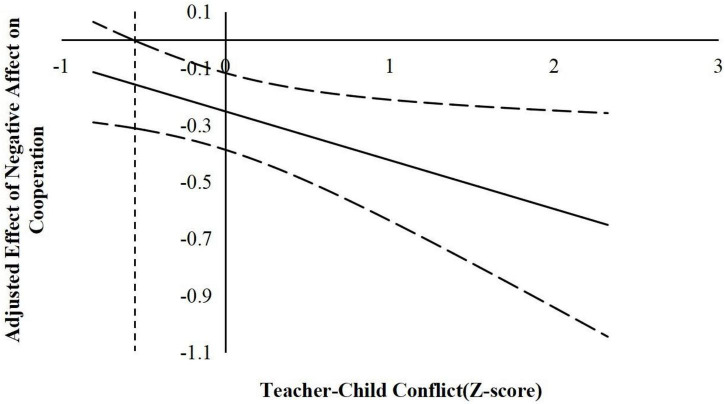
The moderating effect of teacher-child conflict in the prediction of children’s negative affect on cooperation. The dashed vertical line indicates the point along the teacher-child conflict in which the negative affect regression coefficient transitions from significance (right of the dashed vertical line) to non-statistical significance (left of the dashed vertical line). The value of the dashed vertical line was –0.56.

**FIGURE 4:**
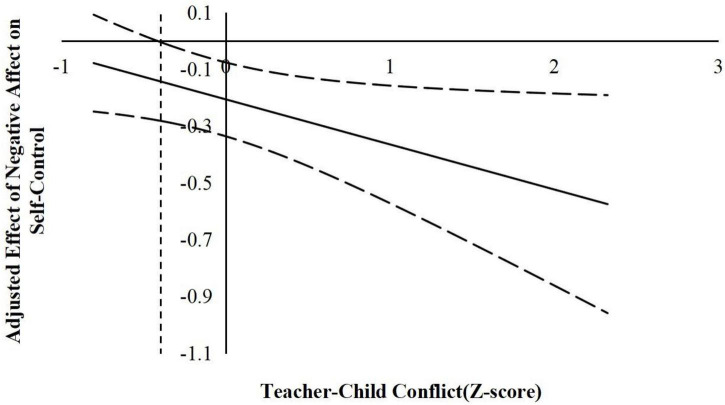
The moderating effect of teacher-child conflict in the prediction of children’s negative affect on self-control. The dashed vertical line indicates the point along the teacher-child conflict in which the negative affect regression coefficient transitions from significance (right of the dashed vertical line) to non-statistical significance (left of the dashed vertical line). The value of the dashed vertical line was 0.42.

**FIGURE 5:**
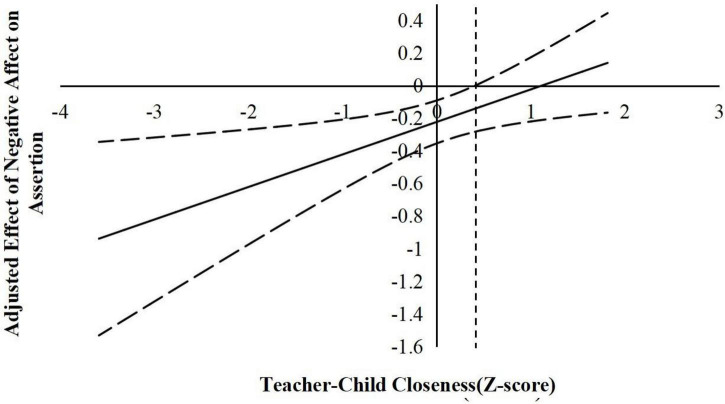
The moderating effect of teacher-child closeness in the prediction of children’s negative affect on the assertion. The dashed vertical line indicates the point along the teacher-child closeness in which the negative affect regression coefficient transitions from significance (left of the dashed vertical line) to non-statistical significance (right of the dashed vertical line). The value of the dashed vertical line was 0.41.

**FIGURE 6:**
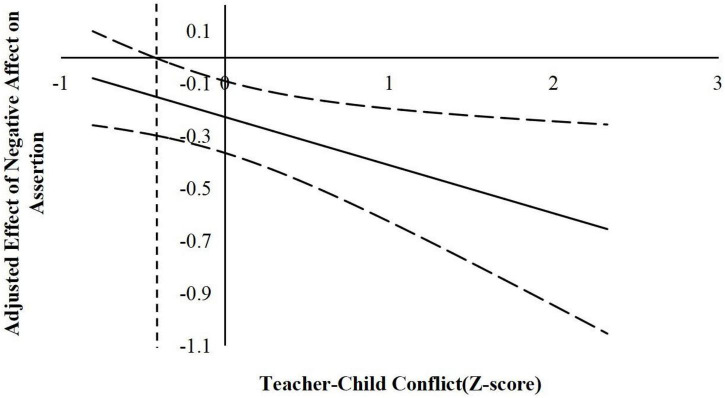
The moderating effect of teacher-child conflict in the prediction of children’s negative affect on the assertion. The dashed vertical line indicates the point along the teacher-child conflict in which the negative affect regression coefficient transitions from significance (right of the dashed vertical line) to non-statistical significance (left of the dashed vertical line). The value of the dashed vertical line was –0.43.

According to suggestions by Hayes and Matthes (2009), the following simple slope analyses used the J–N technique to further explain the interaction effects. This technique allowed us to estimate a region of significance for the simple slope of a predictor conditioned on the value of the continuous moderator. The results are shown in Figures 2–6.

For the prediction of cooperation. As indicated in Figure 2, when the teacher–child closeness level was lower than 0.53 *SD*, negative affect was significantly negatively associated with children’s cooperation. However, when the teacher–child closeness level was higher than 0.53 *SD*, negative affect was no longer associated with cooperation. As shown in Figure 3, when the teacher–child conflict level was higher than −0.56 *SD*, negative affect was significantly negatively associated with children’s cooperation. However, when the teacher–child conflict level was lower than −0.56 *SD*, negative affect was no longer associated with cooperation.

For the prediction of self-control. As indicated in Figure 4, when the teacher–child conflict level was higher than −0.42 *SD*, negative affect was significantly negatively associated with children’s self-control. However, when the teacher–child conflict level was lower than −0.42 *SD*, negative affect was no longer associated with self-control.

For the prediction of assertion. As indicated in Figure 5, when the teacher–child closeness level was lower than 0.41 *SD*, negative affect was significantly negatively associated with children’s assertion. However, when the teacher–child closeness level was higher than 0.41 *SD*, negative affect was no longer associated with assertion. As indicated in Figure 6, when the teacher–child conflict level was higher than −0.43 *SD*, negative affect was significantly positively associated with children’s assertion. However, when the teacher–child conflict level was lower than −0.43 *SD*, negative affect was no longer associated with assertion.

## Discussion

Although previous studies have demonstrated the associations between different components of children’s temperament and social-emotional development, as well as provided meaningful information about the two variables in different contexts (Davies et al., 2021; Zhu J. et al., 2021), the effect of negative affect on children’s social skills has been less explored, and the possible moderating effect of teacher–child relationships remains unexplored. Thus, the primary goal of this study was to examine the associations between preschool children’s negative affect and social skills, and the moderating effect of the teacher–child relationship. The results showed that teacher–child closeness and conflict played moderating roles in the relationship between children’s negative affect and the three indices of social skills (cooperation, self-control, and assertion). The results revealed the underlying mechanisms of preschool children’s negative affect on their social skills.

### The relationships between preschool children’s negative affect and social skills

After controlling for T1 social skills and gender, preschoolers’ negative affect significantly and negatively predicted cooperation, self-control, and assertion after 1 year, which is consistent with a previous study about infants (Jones et al., 2002). The results are also consistent with the direct linear effects model of child development (Sanson et al., 2004); that is, some extreme temperaments can lead to adverse developmental outcomes in children. Children with a high level of negative affect are often characterized by irritability, poor ability to resist setbacks, easy frustration, and lack of comfort. Therefore, they also tend to show more problem behaviors than their counterparts (Taylor et al., 2014), which is not conducive to the development of positive social relationships and hinders their opportunities to learn social skills. Particularly, in preschool age, most children gradually adapt to their new social life and learn how to develop positive peer relationships. A high frequency of peer interactions provides them with more opportunities to practice social skills (Wang et al., 2020). However, a high level of negative affect hinders children’s social interactions. For example, Zhu et al. (2019) found that shy infants showed more negative emotions such as anxiety and depression, and these children generally had lower levels of social skills than did their counterparts. A study of preschool children with social avoidance also found that children who frequently avoided social interaction also showed poor social adaptation (Zhu J. et al., 2021).

High negative affect among children may also influence the parent–child relationship and lead to negative parenting behaviors (Stone et al., 2016), which further aggravates children’s lack of social skills. For example, studies have shown that infants with high negative emotions and low effort control will lead mothers to experience high parenting pressure in parent–child interactions, perhaps even making it dysfunctional, which will affect the development of children’s social skills (Mash and Johnston, 1990; McBride et al., 2002).

Finally, high negative affect influences not only children’s social relationships but also their cognition of the social environment, thus resulting in the avoidance of social interactions. For example, children with a high level of fear usually attribute their emotions to unpredictable factors beyond their control (Lerner and Keltner, 2001). Therefore, when they face unfamiliar environments, they are often less adaptable. They also shy away from social interactions owing to their fear of peer rejection. However, negative emotions such as depression and irritability affect their cognition with respect to social relationships. They believe that their parents do not love them and restrict their actions, and they tend to form avoidant attachment relationships, thus limiting the development of social skills.

### The moderating effect of teacher–child relationship

The teacher–child relationship moderated the associations between negative affect and social skills (cooperation, self-control, and assertion), which showed that teacher–child closeness buffered the negative prediction of children’s negative affect on their social skills, and teacher–child conflict intensified the negative prediction of negative affect on social skills. The results also supported the differential susceptibility model and developmental systems theory concerning children’s temperament and their development (Belsky, 1997; Myers and Pianta, 2008). The differential susceptibility model suggests that children with extreme temperaments are more likely to be positively affected by a favorable environment as compared to their counterparts. Children with a high level of negative affect are also more sensitive to the conflicted teacher–child relationship in kindergarten and are also more susceptible to the positive influence of close teacher–child relationships compared to their counterparts. The developmental systems theory suggests that an individual’s development is depicted as a dynamic process influenced by the interaction between individual traits and the external environment, and a person can shape and be shaped by the environment. Consistent with the theory, the results found that negative affect and teacher–child relationships influenced preschool children’s social skills. Negative affect as one of the most important individual characteristics has different effects on children’s social skills per distinct teacher–child relationship.

The protective role of the teacher–child closeness was also consistent with the attachment theory. Preschool teachers are a key part of kindergarteners’ lives, and a positive teacher–child relationship provides a secure base for children with a high level of negative affect to freely explore their surroundings and interact with others, which is crucial for the development of their social skills (Wu et al., 2018; Baardstu et al., 2022). However, the goodness-of-fit theory emphasizes that only the best match between the external environment and children’s temperament can promote improved development (Thomas and Chess, 1977). Similarly, a good match between temperament and teacher–child relationship is crucial to promoting the development of children’s social skills. Therefore, a close teacher–child relationship could effectively compensate for a high level of negative affect. Teachers’ active attention and equal acceptance can promote these children to participate in activities, help them cope with difficult social situations effectively, and enhance their ability to handle negative emotions. In addition, a close teacher–child relationship also affects peers’ views and attitudes toward children with a high negative affect, which is conducive to promoting positive peer interactions (Wang et al., 2016).

## Limitations and implications

Several limitations exist in this study. First, the data were all self-reported by mothers and teachers, and there was a lack of data from children. In the future, researchers should also collect preschool children’s opinions of teacher–child relationships and social skills through interviews with the children. Second, according to the theoretical framework, family environmental factors may also have an interaction effect on children’s temperament, while our study only paid attention to the teacher–child relationships. Future studies should include family environment factors to examine the interaction between children’s temperament and those factors on their social and emotional competence. Third, the data were collected in Shanghai, the largest city in China; thus, the generalizability of the findings is limited.

Despite these limitations, the findings have important theoretical and practical implications. First, the results elucidate the links between negative affect and social skills and the role of the teacher–child relationship. The results demonstrated and expanded relevant theory models, such as the attachment theory, the developmental systems theory, and the differential susceptibility model, which help clarify the interaction effect of temperament and external environment on children’s social and emotional development. Second, depending on the context, teacher–child relationships had a protective or an aggravating effect on the development of social skills among children with a high level of negative affect. Therefore, a harmonious and close teacher–child relationship can help children to learn more social skills through positive teacher–child interactions. Teachers should pay more attention to children with a high level of negative affect and improve the quality of their interactions with these children. In addition, schools can implement intervention programs aimed at improving the quality of teacher–child relationships, to help preschool teachers better interact with children and form positive teacher–child relationships. For example, Driscoll and Pianta (2010) developed an intervention program named “Banking Time,” which aimed to change the teacher–child interaction model (e.g., observe children’s behaviors, recognize children’s emotions, and describe children’s behaviors and emotions). This could improve teacher–child relationship quality, foster children’s social skills, and reduce problem behaviors in the classroom (Driscoll and Pianta, 2010; LoCasale-Crouch et al., 2018).

## Conclusion

This study revealed an association between preschool children’s negative affect and social skills (cooperation, self-control, and assertion). A higher level of negative affect may lead to a lower level of social skills. The teacher–child relationship moderated the association between negative affect and children’s social skills, with closeness buffering the prediction of negative affect on children’s social skills, while conflict intensifying the prediction of negative affect on children’s social skills.

## Data availability statement

The raw data used during the current study will be made available by the corresponding author upon reasonable request.

## Ethics statement

The studies involving human participants were reviewed and approved by Shanghai Normal University. Written informed consent to participate in this study was provided by the participants’ legal guardian/next of kin. Written informed consent was obtained from the individual(s), and minor(s)’ legal guardian/next of kin, for the publication of any potentially identifiable images or data included in this article.

## Author contributions

YW and YL conceived of the presented idea. YW analyzed the data, wrote up the “Materials and methods” and “Results” section, and verified the analytical methods. YW and DH wrote up the first draft. YL, YT, and LZ supervised the findings of this work. All authors discussed the results, contributed to the final manuscript.
